# Supplementary material for: Complex carbohydrate utilization by gut bacteria modulates host food consumption
Source: Nat Commun. 2025 Sep 25;16:8408. doi: 10.1038/s41467-025-63372-8 (PMC12462444; doi:10.1038/s41467-025-63372-8)
Supplement: Supplementary file 1 — Supplementary Information [file 41467_2025_63372_MOESM1_ESM.pdf]

Supplementary Information for

Complex carbohydrate utilization by gut bacteria modulates host food consumption

Kristie B. Yu *et al.*

Corresponding authors: Kristie B. Yu, kbyu@mednet.ucla.edu,

Elaine Y. Hsiao, ehsiao@g.ucla.edu

This PDF file includes:

Supplementary Figures 1 to 7

Supplementary Tables S1 and S2

Raw input data for each graph of all figures are provided in the Source Data file.

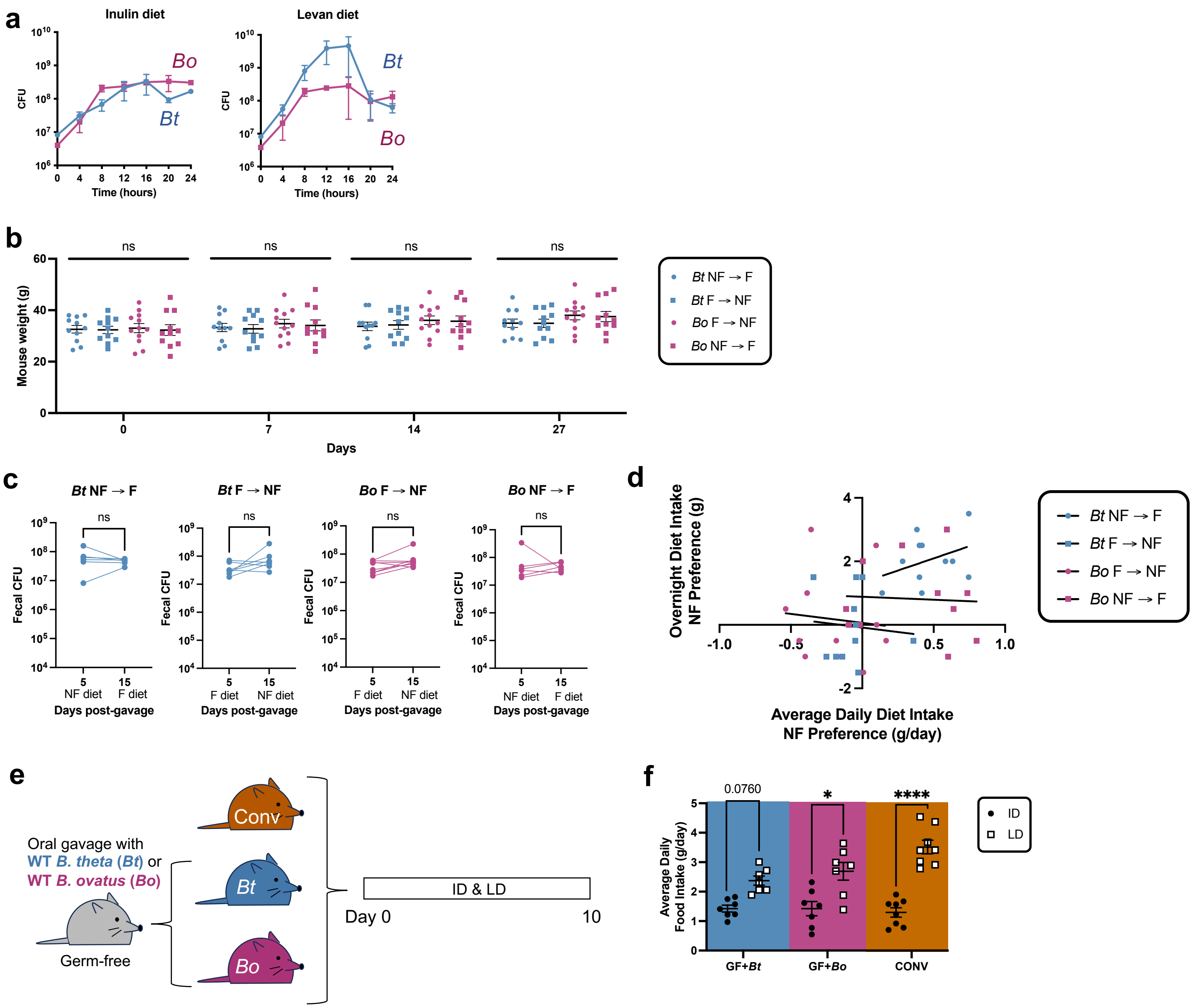

Supplementary Figure 1: Body weight and bacterial load in *Bacteroides* mono-colonized mice fed diets containing non-fermentable or fermentable fructan.

a) Growth curves of *Bt* and *Bo* in minimal media containing 5% inulin diet (ID) or levan diet (LD). Each dot represents mean of 3 biological replicates, error bars represent SEM. CFU = colony forming unit. **b**) Mouse weights during the sequential feeding experiment. ANOVA column (group) factor p-value = 0.8218, ANOVA time factor p-value < 0.0001. **c**) Mouse fecal bacterial load during the sequential feeding experiment, separated by bacteria and diet order. The diets that mice were consuming are listed underneath the x-axis. p-values (from left to right) = 0.3385, 0.2007, 0.1603, 0.5247. **d**) Linear correlation plot for NF "preference" (NF intake minus F intake) in average daily diet intake and overnight diet intake. Slopes were not significantly non-zero (p-value = 0.2662 for *Bt* NF→F, 0.7949 for *Bt* F→NF, 0.7620 for *Bo* F→NF, and 0.8784 for *Bo* NF→F). **e**) Experimental schematic for non-habituated, non-sequential feeding of ID and LD. Germ-free mice that were colonized with *Bt* or *Bo* (GF+*Bt* or GF+*Bo*) or mice with conventional microbiota (CONV) ate both ID and LD *ad libitum* for 10 days. **f**) Average daily diet intake for GF+*Bt*, GF+*Bo*, or CONV mice. p-values (from left to right) = 0.0760, 0.0133, <0.0001. ANOVA interaction p-value = 0.07.

Error bars for b, f represent mean ± SEM. Data for b-d combined from three independent experiments. For b and d, n = 11 for *Bt* NF → F, 11 for *Bt* F → NF, 12 for *Bo* F → NF, 11 for *Bo* NF → F. For c, n = 6 for *Bt* NF → F, 7 for *Bt* F → NF, 8 for *Bo* F → NF, 6 for *Bo* NF → F. For f, n = 7 for GF+*Bt*, 7 for GF+*Bo*, 8 for CONV. For b, 2-way ANOVA with matched row (time) measures, comparing means across rows and columns, and Sidak's corrections were performed. For c, paired parametric t-tests (two-tailed) were performed. For d, simple linear regressions were performed. For f, 2-way ANOVA with matched column measures, comparing means across rows, and Sidak's corrections were performed. ns = p-value > 0.10; * = p-value < 0.05; **** = p-value < 0.0001. F = fermentable diet, NF = non-fermentable diet.

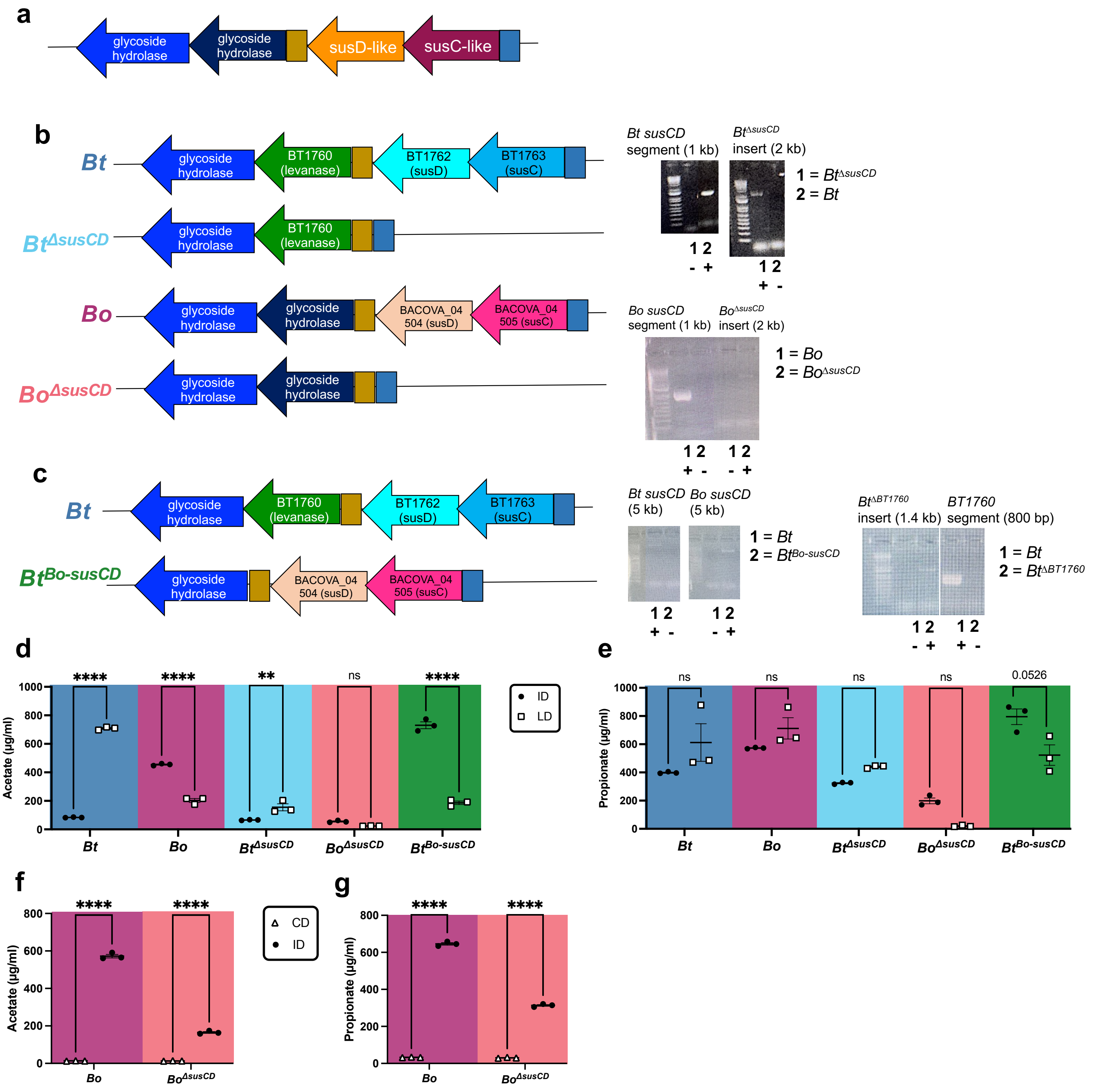

Supplementary Figure 2: Validation of engineered *Bacteroides* in growth assays and production of short chain fatty acids.

a) Schematic of fructan *sus* operon in both *Bt* and *Bo*. One of the glycoside hydrolases in *Bt* is specific for linkages in levan, called levanase (BT1760). *susC-* and *susD-like* genes from this operon are henceforth referred to as *susC* and *susD* or *susCD* genes. **b**) Schematic of fructan *sus* operons for Δ*susCD* bacterial strains with confirmatory PCR gels showing appropriate deletion. **c**) Schematic of fructan *sus* operon for inulin-utilizing *B. thetaiotomicron* (*Bt*$^{Bo\text{-}susCD}$) with confirmatory PCR gels showing appropriate insertion and deletion. **d**) Acetate produced in supernatants of bacteria grown for 24 hours in minimal media containing 5% inulin diet (ID) or levan diet (LD). p-values (from left to right) = <0.0001, <0.0001, 0.0071, 0.5806, <0.0001. ANOVA interaction p-value < 0.0001. **e**) Propionate produced in supernatants of bacteria grown for 24 hours in minimal media containing 5% ID or LD. p-values (from left to right) = 0.1584, 0.5281, 0.6937, 0.2915, 0.0526. ANOVA interaction p-value = 0.0094. **f**) Acetate produced in supernatants of bacteria grown for 24 hours in minimal media containing 5% cellulose diet (CD) or ID. p-values (from left to right) = <0.0001, <0.0001. ANOVA interaction p-value < 0.0001. **g**) Propionate produced in supernatants of bacteria grown for 24 hours in minimal media containing 5% CD or ID. p-values (from left to right) = <0.0001, <0.0001. ANOVA interaction p-value < 0.0001.

For b-c, PCR gel images were cropped and merged for easier visualization. For d-g, n = 3 each, and error bars represent mean ± SEM. For d-g, 2-way ANOVA with matched column measures, comparing means across rows, and Sidak's corrections were performed. ns = p-value > 0.10, * = p-value < 0.05; ** = p-value < 0.01; **** = p-value < 0.0001. ID = inulin diet, LD = levan diet, CD = cellulose diet.

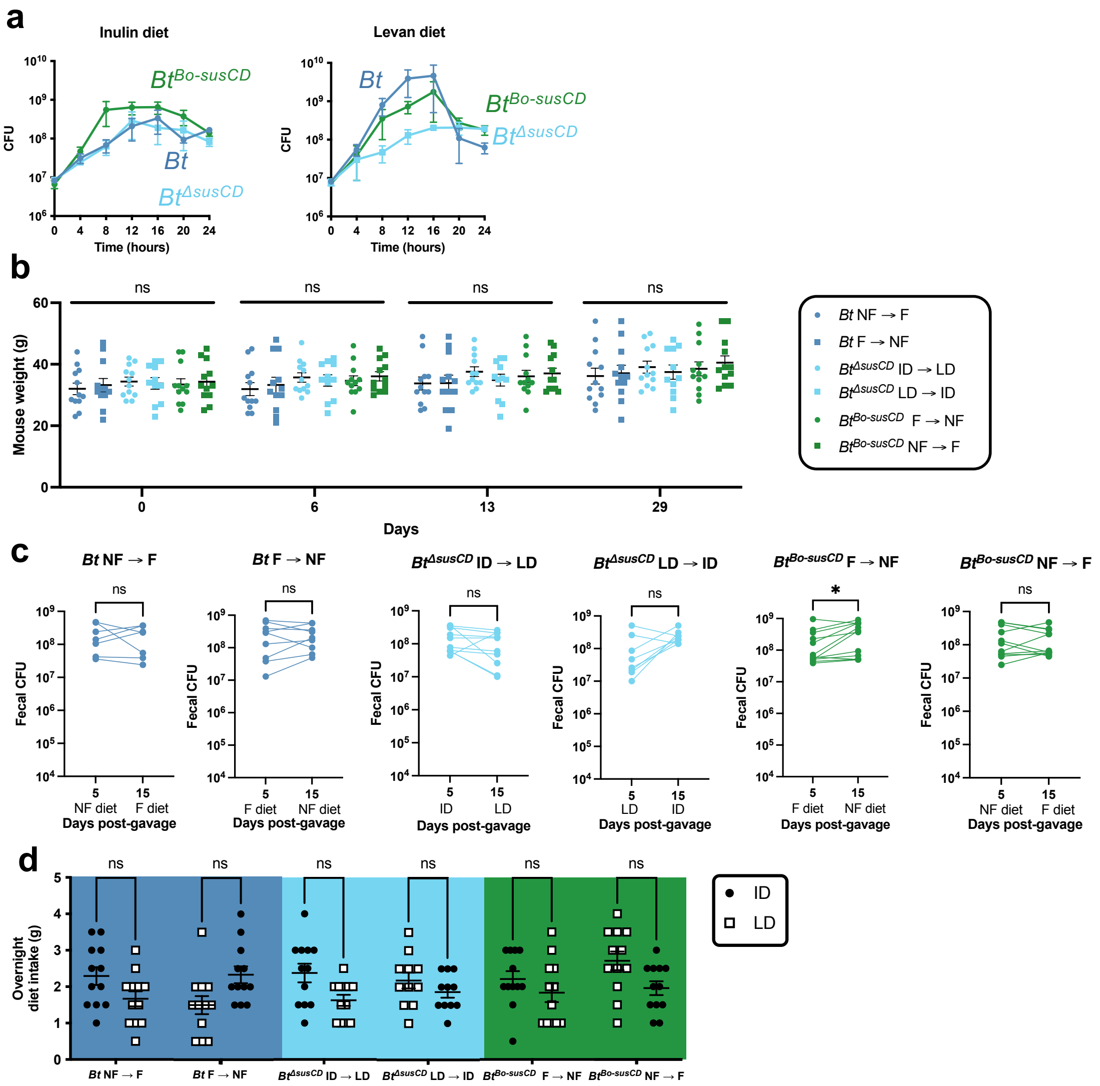

Supplementary Figure 3: Body weight, bacterial load, and overnight diet intake in mice mono-colonized with wildtype or engineered *B. thetaiotaomicron*.

a) Growth curves of *Bt*, $Bt^{\Delta susCD}$, and $Bt^{Bo\text{-}susCD}$ in minimal media containing 5% inulin diet (ID) or levan diet (LD). Each dot represents mean of 3 biological replicates, error bars represent SEM. CFU = colony forming unit. **b**) Mouse weights during the sequential feeding experiment.
ANOVA column (group) factor p-value = 0.7963, ANOVA time factor p-value < 0.0001. **c**) Mouse fecal bacterial load during the sequential feeding experiment, separated by bacteria and diet order. The diets that mice were consuming are listed underneath the x-axis. p-values (from left to right) = 0.7917, 0.7715, 0.2305, 0.1637, 0.0338, 0.5831. **d**) Overnight diet intake from days 30-31 for mice colonized with *Bt*, $Bt^{\Delta susCD}$, and $Bt^{Bo\text{-}susCD}$ by diet order. p-values (from left to right) = 0.4122, 0.1287, 0.2151, 0.9518, 0.8798, 0.2151. ANOVA interaction p-value = 0.012.
Error bars for b, d represent mean ± SEM. Data from b-d are combined from three independent experiments. For b and d, n = 12 for *Bt* NF → F, 12 for *Bt* F → NF, 12 for $Bt^{\Delta susCD}$ ID → LD, 11 for $Bt^{\Delta susCD}$ LD → ID, 12 for $Bt^{Bo\text{-}susCD}$ F → NF, and 12 for $Bt^{Bo\text{-}susCD}$ NF → F. For c, n = 7 for *Bt* NF → F, 9 for *Bt* F → NF, 10 for $Bt^{\Delta susCD}$ ID → LD, 8 for $Bt^{\Delta susCD}$ LD → ID, 12 for $Bt^{Bo\text{-}susCD}$ F → NF, and 11 for $Bt^{Bo\text{-}susCD}$ NF → F. For b, 2-way ANOVA with matched row (time) measures, comparing means across rows and columns, and Sidak's corrections were performed. For c, paired parametric tests (two-tailed) were performed. For d, 2-way ANOVA with matched column measures, comparing means across rows, and Sidak's corrections were performed. ns = p-value > 0.10; * = p-value < 0.05. F = fermentable diet, NF = non-fermentable diet.

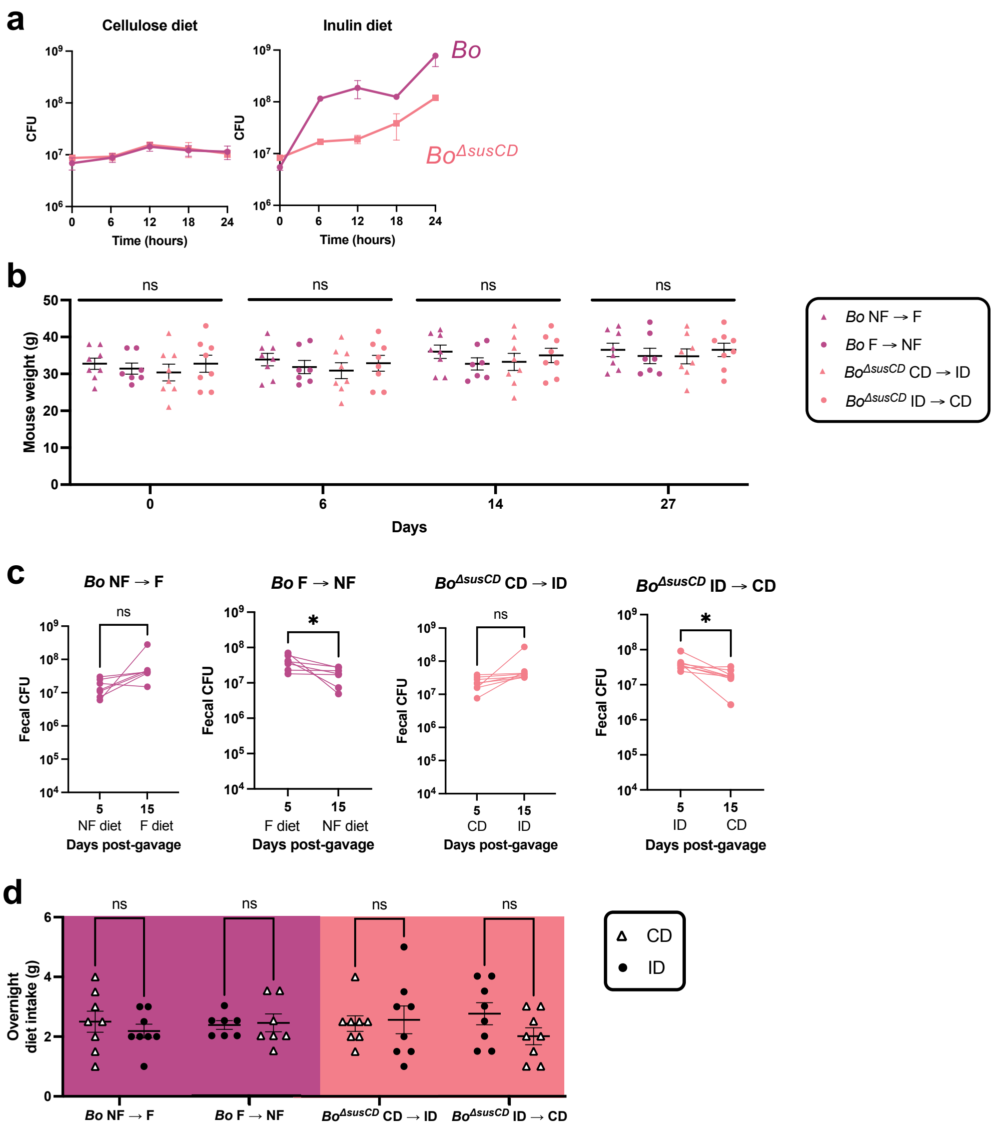

Supplementary Figure 4: Body weight, bacterial load, and overnight diet intake in mice mono-colonized with wildtype or engineered *B. ovatus*.

a) Growth curves of *Bo* and *Bo*$^{\Delta susCD}$ in minimal media containing 5% cellulose diet (CD) or inulin diet (ID). Each dot represents mean of 3 biological replicates, error bars represent SEM. CFU = colony forming unit. **b**) Mouse weights during the sequential feeding experiment. ANOVA column (group) factor p-value = 0.7633, ANOVA time factor p-value < 0.0001. **c**) Mouse fecal bacterial load during the sequential feeding experiment, separated by bacteria and diet order. The diets that mice were consuming are listed underneath the x-axis. p-values (from left to right) = 0.1725, 0.0351, 0.1800, 0.0213. **d**) Overnight diet intake from days 30-31 for mice colonized with *Bo* and *Bo*$^{\Delta susCD}$ by diet order. p-values (from left to right) = 0.9112, 0.9997, 0.9969, 0.2817. ANOVA interaction p-value = 0.3241.

Error bars for b, d represent mean ± SEM. Data from b-d are combined from two independent experiments. For b, d, n = 8 for *Bo* NF→ F, 7 for *Bo* F → NF, 8 for *Bo*$^{\Delta susCD}$ CD → ID, and 8 for *Bo*$^{\Delta susCD}$ ID → CD. For c, n = 7 for *Bo* NF → F, 7 for *Bo* F → NF, 8 for *Bo*$^{\Delta susCD}$ CD → ID, and 8 for *Bo*$^{\Delta susCD}$ ID → CD. For b, 2-way ANOVA with matched row (time) measures, comparing means across rows and columns, and Sidak's corrections were performed. For c, paired parametric t-tests (two-tailed) were performed. For d, 2-way ANOVA with matched column measures, comparing means across rows, and Sidak's corrections were performed. ns = p-value > 0.10; * = p-value < 0.05. F = fermentable diet, NF = non-fermentable diet.

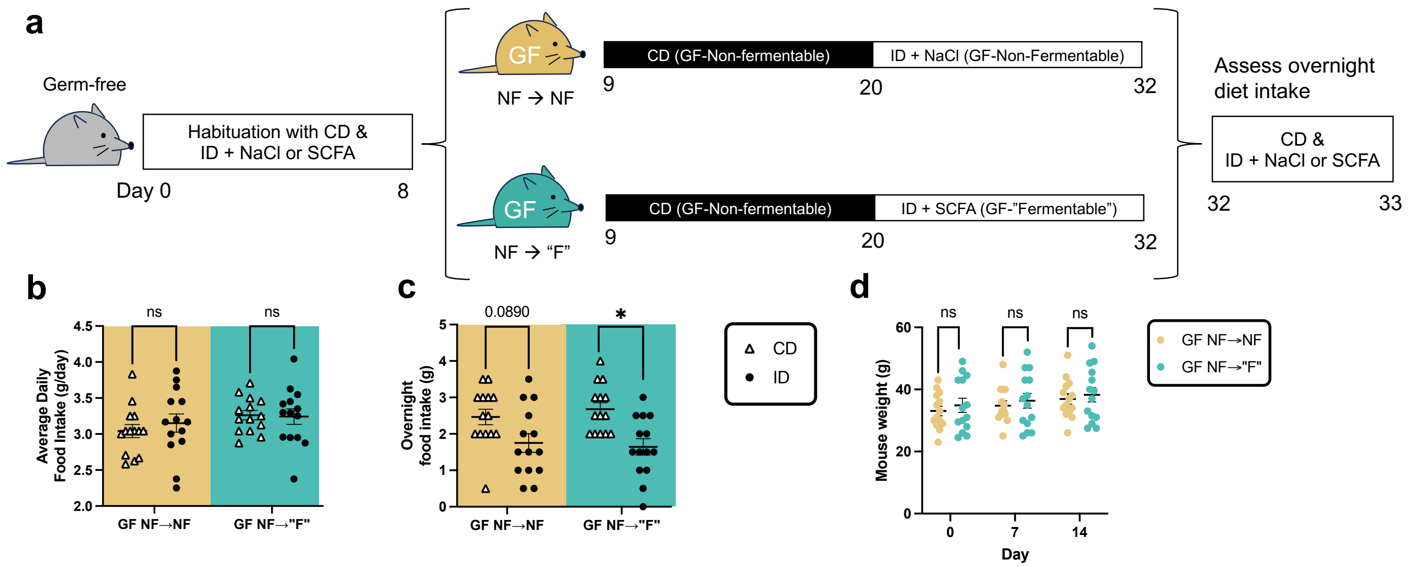

Supplementary Figure 5: Dietary SCFA supplementation is not sufficient to alter host dietary consumption.

a) Experimental schematic for sequential feeding with SCFA supplementation**.** Germ-free mice were fed 5% cellulose diet (CD) and 5% inulin diet (ID) supplemented with NaCl (sodium chloride 92.5 mmol/g) as a control or SCFA (acetate 67.5 mmol/g and propionate 25 mmol/g)**.** Mice were habituated with both diets for 8 days, then switched to single diets: CD *ad libitum* for 12 days and then ID + NaCl or ID + SCFA for 12 days. The mice were then provided both diets in their cage overnight. **b**) Average daily diet intake during single diet feeding for GF NF→NF and GF NF→"F" mice. p-values (from left to right) = 0.5098, 0.9767. ANOVA interaction p-value = 0.3840. **c**) Overnight diet intake from days 32-33 for GF NF→NF and GF NF→"F" mice. p-values (from left to right) = 0.0890, 0.0105. ANOVA interaction p-value = 0.5098. **d)** Mouse weights during the sequential feeding experiment. ANOVA column (group) factor p-value = 0.5753, ANOVA time factor p-value < 0.0001.
Error bars for b-d represent mean ± SEM. Data from b-d are combined from two independent experiments. For b-d, n = 14 for GF NF→NF and 14 for GF NF→"F". For b-c, 2-way ANOVA with matched column measures, comparing means across rows, and Sidak's corrections were performed. For d, 2-way ANOVA with matched row (time) measures, comparing means across rows and columns, and Sidak's corrections were performed. ns = p > 0.10; * = p-value < 0.05. "F" = "fermentable" diet (ID + SCFA), NF = non-fermentable diet (ID + NaCl).

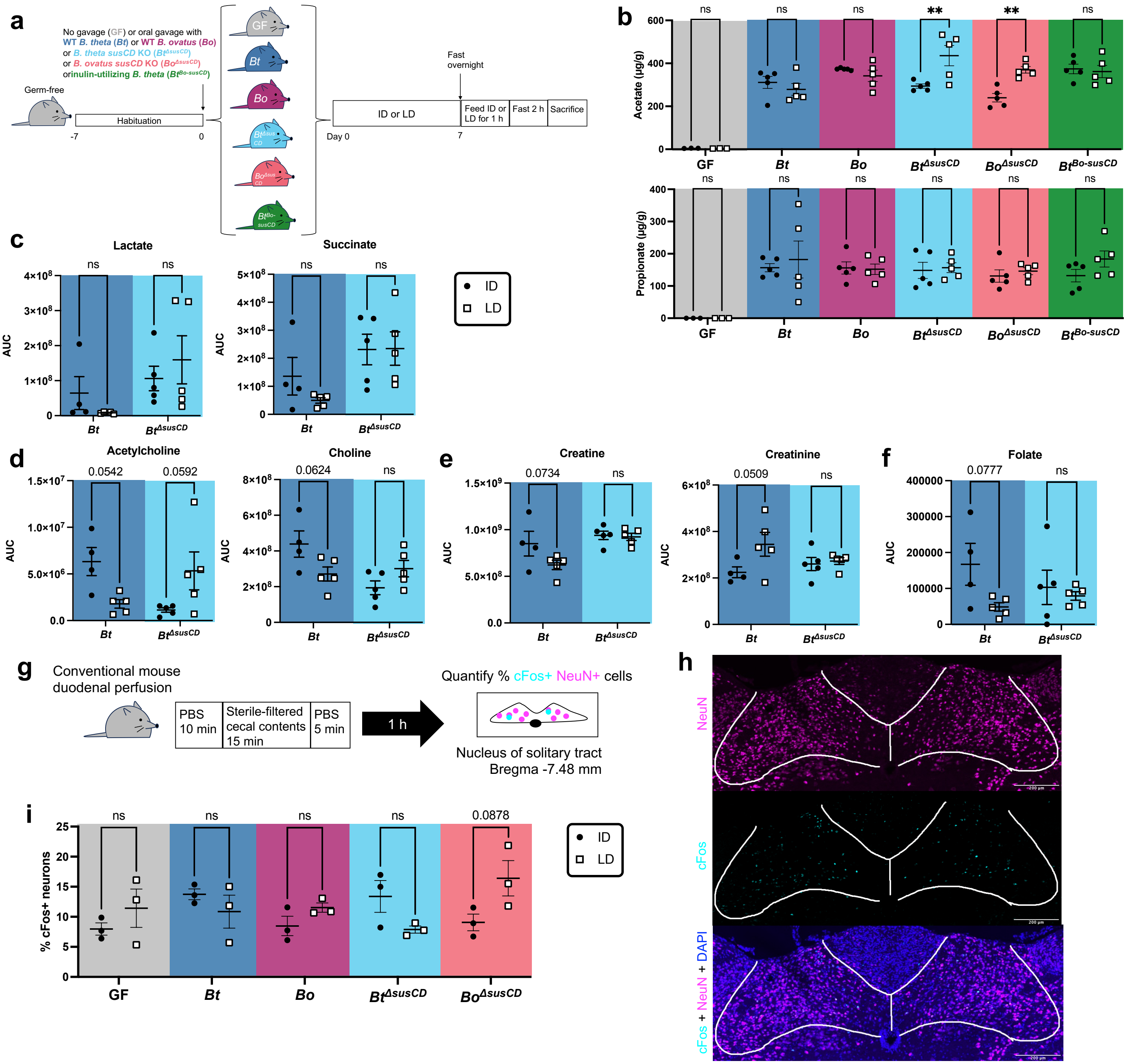

Supplementary Figure 6: Bacterial fructan utilization by *B. thetaiotaomicron* alters metabolite levels in cecal content.

a) Experimental schematic for germ-free mice (GF, no colonization) or mice colonized with with *Bt*, $Bt^{\Delta susCD}$, *Bo*, $Bo^{\Delta susCD}$, and $Bt^{Bo\text{-}susCD}$ and fed either 5% inulin diet (ID) or levan diet (LD) for 7 days. Mice were habituated for 7 days outside gnotobiotic isolator before colonization. On day 7, mice were fasted overnight before being fed ID or LD for 1 hour, and after 2 hours, they were sacrificed to collect cecal content. **b**) Acetate and propionate levels in cecal content of GF or colonized mice. p-values (from left to right) for acetate = >0.9999, 0.9260, 0.9357, 0.0010, 0.0029, 0.9995; p-values (from left to right) for propionate = >0.9999, 0.9755, >0.9999, >0.9999, 0.9985, 0.5894. ANOVA interaction p-value = 0.0005 for acetate and 0.8882 for propionate. **c**) Lactate and succinate levels in cecal content of mice colonized with *Bt* or $Bt^{\Delta susCD}$. p-values (from left to right) for lactate = 0.6504, 0.6461; p-values (from left to right) for succinate = 0.4618, 0.9986. ANOVA interaction p-value = 0.2464 for lactate and 0.3967 for succinate. **d**) Acetylcholine and choline levels in cecal content of mice colonized with *Bt* or $Bt^{\Delta susCD}$. p-values (from left to right) for acetylcholine = 0.0542, 0.0592; p-values (from left to right) for choline = 0.0624, 0.2439. ANOVA interaction p-value = 0.0038 for acetylcholine and 0.0129 for choline. **e**) Creatine and creatinine levels in cecal content of mice colonized with *Bt* or $Bt^{\Delta susCD}$. p-values (from left to right) for creatine = 0.0734, 0.9818; p-values (from left to right) for creatinine = 0.0509, 0.9549. ANOVA interaction p-value = 0.1437 for creatine and 0.1278 for creatinine. **f**) Folate levels in cecal content of mice colonized with *Bt* or $Bt^{\Delta susCD}$. p-values (from left to right) = 0.0777, 0.8676. ANOVA interaction p-value = 0.2105. **g**) Schematic of experimental set up: Mice with conventional microbiome under isofluorane anesthesia had a catheter inserted into their gastroduodenal junction, and fluids were perfused into the duodenum out through distal intestine. Mice were first perfused with phosphate-buffered saline (PBS) for a 10 min baseline period, then with sterile-filtered pooled cecal contents from GF or colonized mice fed fructan diet in **a** for 15 min, followed by a 5 min PBS flush. Mice were then statically incubated for 1 hour before collecting their brains. Brains were sectioned coronally for the nucleus of solitary tract (NTS), and medial NTS sections (around Bregma -7.48) were stained for neurons (NeuN, magenta) and early activation marker, cFos (cyan). **h**) Representative image of NTS in a mouse with perfused *Bt*+ID cecal contents. Scale bar for all panels is 200 μm. **i)** Percentage of cFos-positive neurons out of total neurons in NTS of mice perfused with cecal contents of GF or colonized mice fed ID or LD. Each dot represents the average of two technical replicates. p-values (from left to right) = 0.7450, 0.8577, 0.8287, 0.2950, 0.0878. ANOVA interaction p-value = 0.0308.
Error bars for b-f, i represent mean ± SEM. For b, n = 3 for GF and n = 5 for *Bt*, $Bt^{\Delta susCD}$, *Bo*, $Bo^{\Delta susCD}$, and $Bt^{Bo\text{-}susCD}$. For c-f, n = 4 for Bt+ID, 5 for BT+LD, 5 for $Bt^{\Delta susCD}$+ID, and 5 for $Bt^{\Delta susCD}$+LD. For I, n = 3 for all groups. For b-f and i, 2-way ANOVA comparing means across rows and Sidak's corrections were performed. ns = p-value > 0.10; ** = p-value < 0.01. ID = inulin diet, LD = levan diet.

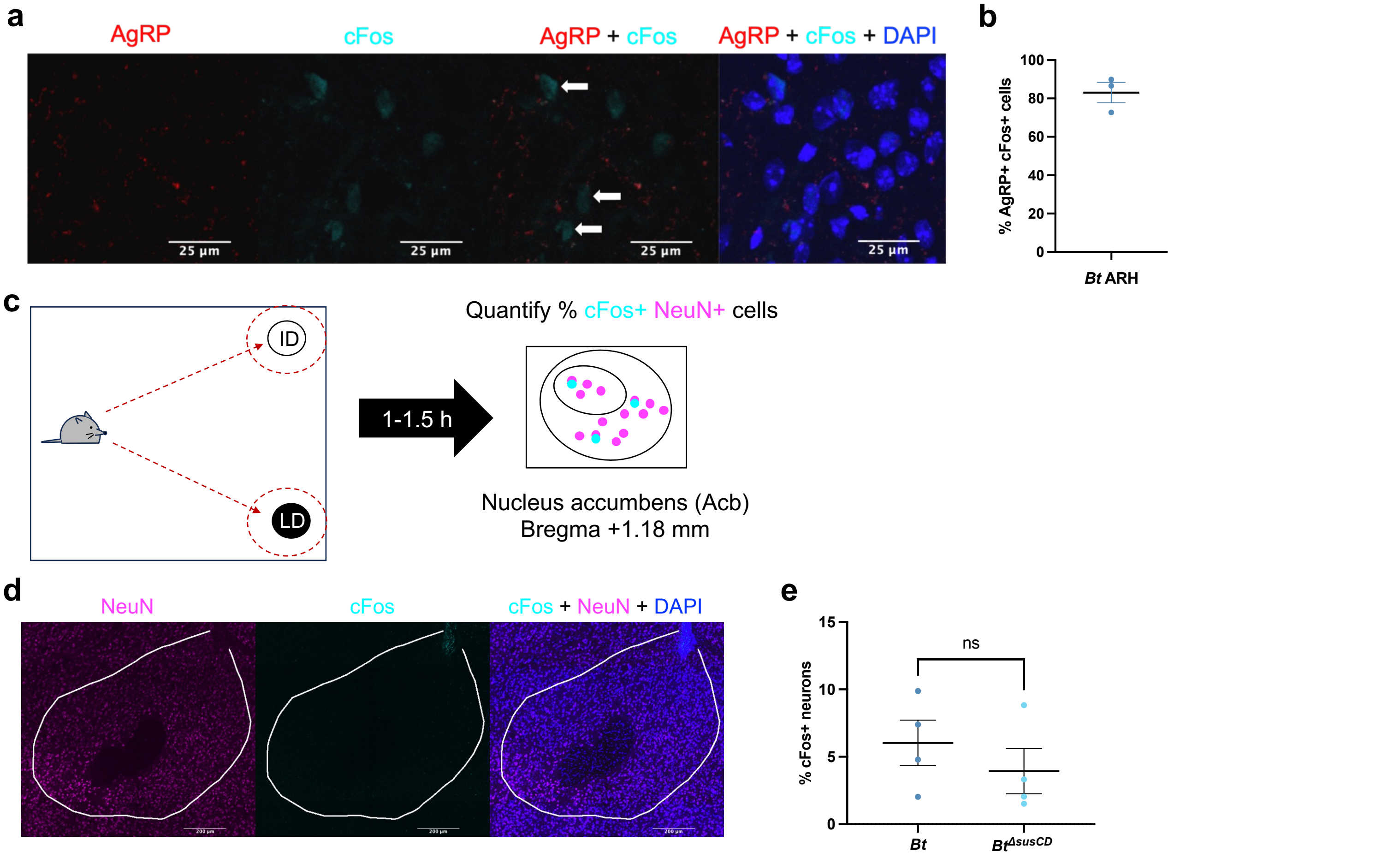

Supplementary Figure 7: Quantification of cFos activation in AgRP+ cells in arcuate nucleus of hypothalamus and neurons in nucleus accumbens.

a) Representative images of AgRP-positive cFos-positive cells in mice colonized with *B. thetaiotaomicron* (*Bt*). White arrows show co-localization between AgRP (red) and cFos (cyan). Scale bar for all panels is 25 µm. **b)** Percentage of AgRP-positive cFos-positive cells out of cFos-positive cells in arcuate nucleus of hypothalamus (ARH) of mice colonized with *Bt* (n = 3 mice). Each dot represents the average of two technical replicates. **c**) Schematic of experimental set up: 1-1.5 hours after the fasting-induced, mice were sacrificed, and their brains collected. Brains were sectioned coronally for nucleus accumbens (ACB), and ACB sections (around Bregma +1.18) were stained for neurons (NeuN, magenta) and early activation marker, cFos (cyan). **d**) Representative images of ACB section in a mouse colonized with *Bt*. Scale bar for all panels is 200 µm. **e**) Percentage of cFos-positive neurons out of total neurons in ARH of mice colonized with *Bt* and *Bt*$^{\Delta susCD}$ (n = 4 mice per group). Each dot represents the average of two technical replicates. Unpaired t-test (two-tailed) with Welch's correction was performed. p-value = 0.4124.
Error bars for b, e represent mean ± SEM. ns = p-value > 0.10.

Supplementary Table S1. Diet formulations

	Cellulose Diet	Inulin Diet	Levan Diet
Catalog number (Inotiv-Teklad)	TD.210524	TD.180786	TD.180785
Ingredient	(g/kg)	(g/kg)	(g/kg)
Casein	200	200	200
DL-Methionine	3	3	3
Vegetable Shortening, hydrogenated (Crisco)	546.518	546.518	546.518
Corn Oil	86.2	86.2	86.2
Vitamin Mix, Teklad	17.8	9.502	9.502
Choline Bitartrate	2.5	2.5	2.5
TBHQ, antioxidant	0.14	0.14	0.14
Mineral Mix, Ca-P Deficient	23.8	28.8	28.8
Calcium Phosphate, dibasic	21.89	21.89	21.89
Calcium Carbonate	6.1	6.1	6.1
Magnesium Oxide	0.35	0.35	0.35
Cellulose	91.702	0	0
Inulin	0	100	0
Levan	0	0	100

63.5% fat, 17.7% protein by weight

Supplementary Table S2. List of cloning primers (5' --> 3')

Linear amplification of pSIE1 plasmid	
pSIE1_F	GGATCCACTAGTTCTAGAG
pSIE1_R	GGATCCTCATAATGCTAATC
B. theta susCD KO (*Bt*$^{\Delta susCD}$) mutant generation	
BTΔ1762_up1000_F	gattagcattatgaggatccTCGTTTCCTCCCCAGCTATTG
BTΔ1762_up1000_R	taaactaatgTAACTAACCATCTTAAAACAAAGAATACAATGAAATC
BTΔ1763_down1000_F	tggttagttaCATTAGTTTAATGTTATTAATTTAAAAGTACGAATTTTCTC
BTΔ1763_down1000_R	gctctagaactagtggatccACGGGAGCATGAAAGTGC
B. ovatus susCD KO (*Bo*$^{\Delta susCD}$) mutant generation	
BOΔ04504_up1000_F	gattagcattatgaggatccATAAGCCCATTCACATGAC
BOΔ04504_up1000_R	caaactaatgTAGTATAAATTCCTTAAATATAAATATGATGAAAATAAAAAG
BOΔ04505_down1000_F	atttatactaCATTAGTTTGATGTTATTAAATTAAAAGTACG
BOΔ04505_down1000_R	gctctagaactagtggatccCAGGTGGAGACCAAAGAC
Inulin-utilizing *B. theta* (*Bt*$^{Bo\text{-}susCD}$) mutant generation	
BTΔ1762_up1000_F	gattagcattatgaggatccTCGTTTCCTCCCCAGCTATTG
BTΔ1762_up1000_R	taagtttaacTAACTAACCATCTTAAAACAAAGAATACAATGAAATC
BO04504_F	tggttagttaGTTAAACTTACCGTATCCG
BO04505_R	taaactaatgCTTAGTATTATGAAGAACAAAAAACTTC
BTΔ1763_down1000_F	taatactaagCATTAGTTTAATGTTATTAATTTAAAAGTACGAATTTTCTC
BTΔ1763_down1000_R	gctctagaactagtggatccACGGGAGCATGAAAGTGC
BTΔ1760_up700_F	gattagcattatgaggatccCCCAGTTCATGTTTCCCC
BTΔ1760_up700_R	agcaacaatgTGATGAAAACTACACCGG
BTΔ1760_down700_F	gttttcatcaCATTGTTGCTTATTCTTTTTATTTAC
BTΔ1760_down700_R	gctctagaactagtggatccATCGATGTCCGAATATATCTC
